# Metabolic rearrangements and intratumoral heterogeneity for immune response in hepatocellular carcinoma

**DOI:** 10.3389/fimmu.2023.1083069

**Published:** 2023-01-25

**Authors:** Fei-Qi Xu, Meng-Meng Dong, Zhi-Fei Wang, Li-Dong Cao

**Affiliations:** ^1^ General Surgery, Cancer Center, Department of Hepatobiliary and Pancreatic Surgery and Minimally Invasive Surgery, Zhejiang Provincial People’s Hospital, Affiliated People’s Hospital, Hangzhou Medical College, Hangzhou, Zhejiang, China; ^2^ The Second School of Clinical Medicine, Zhejiang Chinese Medical University, Hangzhou, China; ^3^ Jilin Provincial Key Laboratory on Molecular and Chemical Genetics, The Second Hospital of Jilin University, Changchun, China

**Keywords:** metabolic rearrangements, intratumoral heterogeneity, immune response, hepatocellular carcinoma, tumor microenvironment

## Abstract

Liver cancer is one of the most common malignant tumors globally. Not only is it difficult to diagnose, but treatments are scarce and the prognosis is generally poor. Hepatocellular carcinoma (HCC) is the most common type of liver cancer. Aggressive cancer cells, such as those found in HCC, undergo extensive metabolic rewiring as tumorigenesis, the unique feature, ultimately causes adaptation to the neoplastic microenvironment. Intratumoral heterogeneity (ITH) is defined as the presence of distinct genetic features and different phenotypes in the same tumoral region. ITH, a property unique to malignant cancers, results in differences in many different features of tumors, including, but not limited to, tumor growth and resistance to chemotherapy, which in turn is partly responsible for metabolic reprogramming. Moreover, the different metabolic phenotypes might also activate the immune response to varying degrees and help tumor cells escape detection by the immune system. In this review, we summarize the reprogramming of glucose metabolism and tumoral heterogeneity and their associations that occur in HCC, to obtain a better understanding of the mechanisms of HCC oncogenesis.

## Background

Each year, the American Cancer Society investigates and publishes the number of new cancer cases in the United States. In 2021, the society projected that 1,898,160 new cancer cases of cancer would be reported in the United States, and that 608,570 cancer patients would die (1). Primary liver cancer is the most frequent cause of cancer death worldwide: its incidence is increasing year on year, and it is the currently the fifth most common type of cancer in the United States. Hepatocellular carcinoma (HCC), the predominant histologic type, accounts for 75%–85% of all liver cancer cases and is the most malignant (2). Intrahepatic cholangiocarcinoma (ICC) is another histologic type of liver cancer, accounting for 10%–15% of cases (3). The risk factors for HCC include chronic hepatitis B virus (HBV) and hepatitis C virus (HCV) infection, obesity, inappropriate aflatoxin intake, smoking, type 2 diabetes mellitus, and alcohol consumption (4). Of note, alcohol consumption is considered the most significant factor for its variables with levels of subjectivity, and the study shows that, even in quantities associated with social drinking, alcohol can potentially increase the risk of HCC development in patients with non-alcoholic steatohepatitis (NASH) or HCV cirrhosis when compared with subjects who do not drink (5). Moreover, early-stage HCC patients are often asymptomatic, but patients with advanced HCC usually miss the best opportunity for treatment, such as surgery or liver transplantation (6). Typical treatments for HCC patients include surgery, radiotherapy, chemotherapy, and targeted molecular therapy (7). For example, sorafenib, a multi-target tyrosine kinase inhibitor (TKI), was first approved by the Food and Drug Administration (FDA) for HCC treatment, and now lenvatinib, sorafenib, and other TKIs are also used in the treatment of HCC (8).

HCC originates from and develops in response to a series of crises, including metabolic, immunological, genetic, and microenvironmental pressures. The effects of these pressures vary over time and space in different states and regions of HCC, promoting the initial progress of HCC as a neoplastic microenvironment exhibiting enormous genetic and phenotypic intratumoral heterogeneity (ITH). Under other growing crises, various tumor cells and stromal components in the tumor microenvironment (TME) develop comparable degrees of ITH (9). Interestingly, genome sequencing has revealed another type of ITH, heterogeneity not in different tumor regions, which is known as spatial ITH, but heterogeneity over time in the same neoplasm, which is known as temporal ITH (10). Significantly, ITH affects both the genetic and epigenetic components of HCC; however, in HCC, these events can be closely connected (active genetic and epigenetic aberrations) or quite separate (stable genome with variable epigenetic modification) (11). In addition, ITH results in extensive metabolic rewiring in HCC throughout oncogenesis and development; this unique feature enables tumor cells to adapt to the neoplastic microenvironment. In addition, ITH leads to the same changes in non-malignant cellular compartments of the TME, such as hepatic stellate cells (HSCs), cancer-associated fibroblasts (CAFs), tumor-infiltrating lymphocytes (TILs), tumor-associated macrophages (TAMs), and dendritic cells (DCs) (12–14). Intriguingly, ITH affects the cellular compartments of TME for HCC malignancy. Conversely, cellular compartments undergoing such spatiotemporal transformation also impact the ITH of HCC (15). For example, although genetic and epigenetic aberrations of cancer cells affect TILs, TILs also influences the proliferation and progress of malignant tumors (16). Classically, ITH was considered a simple binomial state with two extremes (i.e., “on” or “off”) that determine the progression of an aggressive tumor (17). Recently, this description of ITH has been refined, and it is now thought that cellular components with a plastic phenotypic or metabolic state can continuously vary in response to disturbance of the microenvironment (18, 19). It is also thought that ITH can only be tolerated under certain thresholds. Otherwise, the phenotype and metabolic burden of malignant cells and the whole tumoral structure would be impaired (20, 21).

In the 1920s, Otto Warburg was the first to suggest that cancer cells undergo aerobic glycolysis, converting glucose into lactate in the cytoplasm, rather than oxidative phosphorylation (OXPHOS), even under conditions of normoxia (22). This led to the novel idea that tumor cells respond to emerging pressures in their microenvironment by undergoing metabolic rearrangements that promote their survival. This process, which is unique to cancer cells, resembles the Darwinian process of survival of the fittest. Aerobic glycolysis confers a growth advantage on HCC cells, by enhancing glucose uptake, speeding up adenosine triphosphate (ATP) generation, and producing abundant metabolic intermediates and an acidic environment (23). Moreover, increase of aerobic glycolysis in HCC leads to lactate acceleration, which causes a steady reduction in extracellular and intracellular pH. (24) The acidosis induces apoptosis and autophagy in healthy cells; modifies the stromal structure in such a way as to facilitate invasion and migration of HCC cells and metastasis; and also acts as a selection for HCC cells (25). However, OXPHOS also occurs in the mitochondria of HCC cells. According to the reverse Warburg effect, stromal cells such as CAFs support metabolism in cancer cells by releasing glucose-derived metabolites, such as lactate and pyruvate. CAFs utilize glucose in glycolysis and then transport intermediates for OXPHOS into HCC cells (26); OXPHOS is not entirely suppressed in HCC cells. It has been found that ubiquinol–cytochrome c reductase complex assembly factor 3 (UQCC3) is essential in maintaining mitochondrial homeostasis in structure and function, and in regulating OXPHOS activity under hypoxia in HCC cells (27). In an acidic microenvironment, glycolysis is inhibited and therefore, in glycolysis-dominant cells, such as HCC cells, pyruvate is transferred to the mitochondria for OXPHOS (28). The distinct metabolic preference is decided by genetic ITH, which confers plasticity on the metabolic demands of HCC. This transformation of HCC cells is mediated by epigenetic ITH, enablingeasier transition more easily between different cell states (11).

Metabolic reprogramming has been significantly linked with poor prognosis in HCC patients (29). It has consistently been found that genetic and epigenomic ITH affects metabolic rewiring in HCC and predicts poorer outcomes (21). A better comprehension of aerobic glycolysis and ITH and their association with HCC would shed light on the pathogenesis and evolution of HCC. This article aims to summarize the correlation between ITH and metabolic rearrangements (predominantly aerobic glycolysis) and the comprehensive characteristics of the respective biological properties of HCC.

## Heterogeneity in HCC

ITH was first proposed in 1833 by Johannes Muller, 300 years after the microscope was invented (30). This German physiologist utilized microscopy to analyze human tumor samples, imitating methods in phytology, and described tumors as agglomerations of emerging cells, which showed distinct variations among different regions and also differences between tumor cells and adjacent stromal cells (31). ITH has now been unequivocally proven to be present in many cancers. It encompasses dynamic aspects, such as genetic and epigenetic heterogeneity, which can be further classified as transcriptomic, proteomic, and phenotypic heterogeneity, as well as behavioral and immunological heterogeneity, which includes temporal heterogeneity, spatial heterogeneity, and heterogeneity of the TME (metabolic and immunological components), and leads to heterogeneous behavior of tumor cells and changes in the immune responses of immune cells (32).

**Figure 1:**
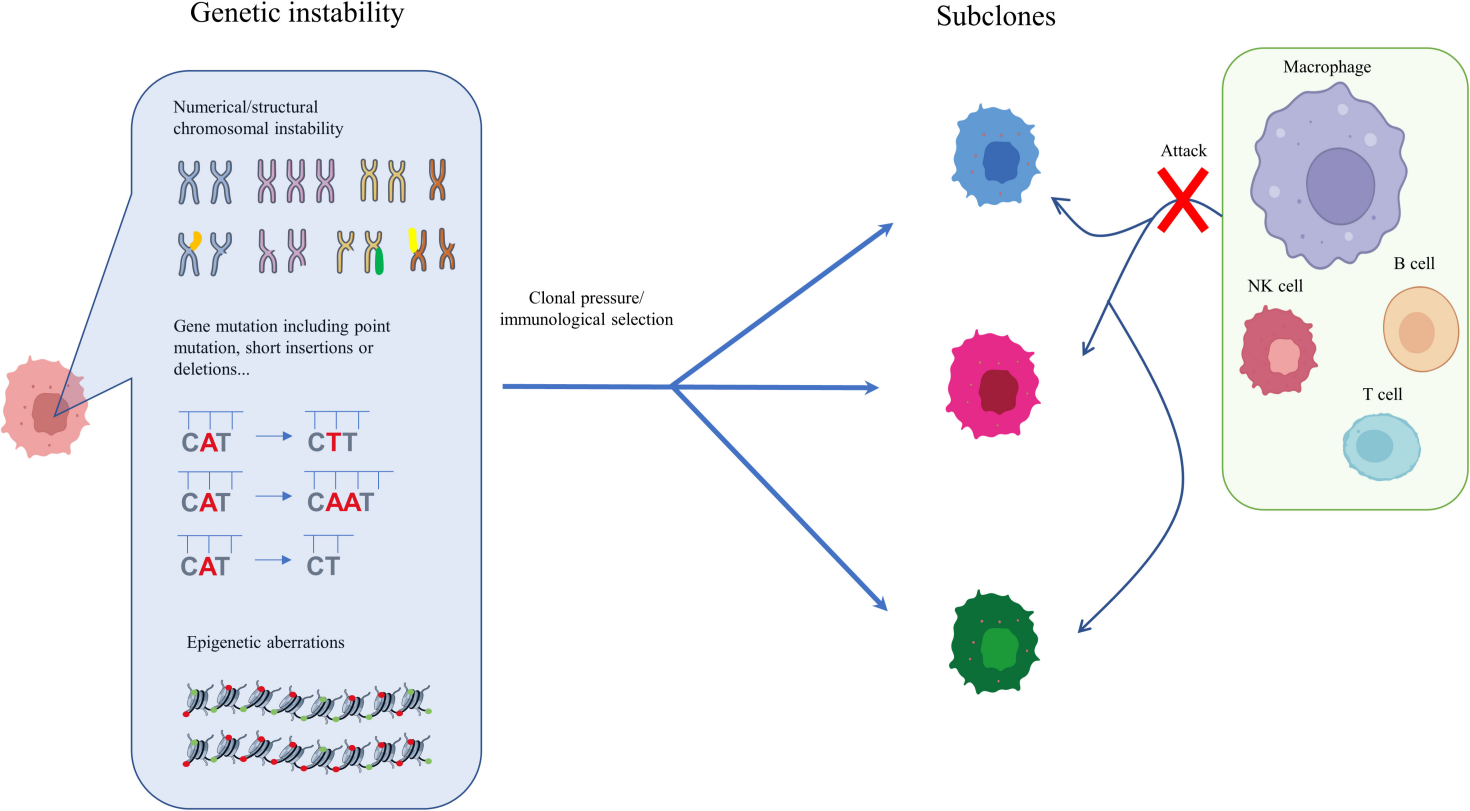
Genetic and epigenetic ITH in HCC. During the initial and subsequent development of HCC, only the fittest clones survive clonal pressure and immunological selection and accumulate in cancer cells. The diversity of surviving cells, which vary in genetic instability, immune escape, and degree of treatment resistance, is considered one reason for the genetic heterogeneity of HCC, which confers on HCC an evolutionary growth advantage. Enhanced genetic ITH normally leads to aggressiveness, immune escape, and resistance to treatment, which is associated poor outcomes and worse prognosis in HCC patients.

Analysis of genetic ITH, involving next-generation sequencing (NGS) studies of the tumoral genetic landscape, have demonstrated that spatiotemporal aberration is caused by genetic instability (20, 33, 34). Genetic instability is reflected in point mutations, short insertions or deletions, copy number variation, and chromosomal alterations, including translocation, deletion, amplification, and aneuploidy (30, 35–37). During the initial development of HCC, only the fittest clones survive clonal pressure and immunological selection and accumulate in cancer cells (38, 39). The diversity of surviving cells, which vary in terms of genetic instability, immune escape, and treatment resistance, is considered the reason for gene heterogeneity in HCC and confers a growth and evolutionary advantage on HCC cells (40–42). High genetic ITH is normally associated with tumor aggressiveness, immune escape, and resistance to treatment, leading to poor outcomes and a worse prognosis for HCC patients (12, 15). Understanding genetic ITH could enable more tailored tumor treatment and could also constitute a negative prognostic marker for HCC patients (43, 44). Epigenetic heterogeneity is regarded as an essential regulator for tumor evolution and development. Generally, epigenetic alterations that induce non-heritable changes in cell clones are reversible, but it is possible that they could be acquired by cell progeny, leading to heritable expression and the progression of cancers (30, 45). One of the most common heritable epigenetic alterations is DNA methylation (46). However, there is no direct evidence that DNA methylation drives cancer initiation in human patients. To date, *in vivo* experiments have shown that targeting the p16Ink4a epimutation drives tumor origination and aggressive development, and attenuates survival time, in mice (47). Furthermore, demethylation of satellite 2 has been found to correlate with chromosome aneuploidy induced by 5-azacytidine (48) (**Figure 1**). DNA methylation has also been shown to be temporally altered in advanced lung cancer (12).

**Figure 2:**
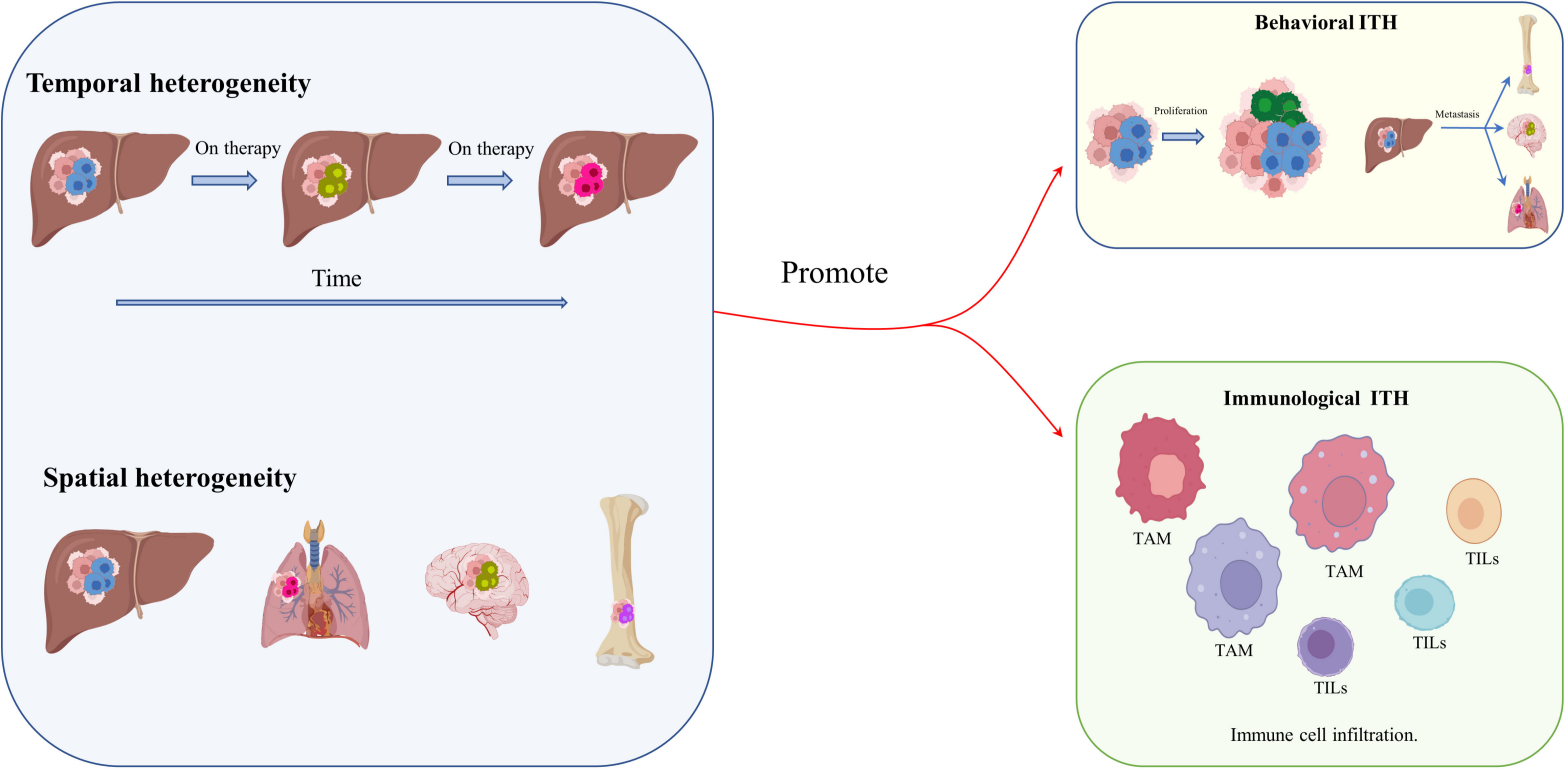
Behavioral and immunological ITH in HCC. Spatiotemporal remodeling of the TME, and especially of its metabolic and immunological components, promotes behavioral and immunological heterogeneity.

As mentioned before, genetic and epigenetic heterogeneity, mainly transcriptomic and proteomic ITH, result in functional heterogeneity, and, together with spatiotemporal remodeling of the TME and changes in metabolic and immunological function, promote behavioral and immunological heterogeneity (49–52) (**Figure 2**). The tumoral ecosystem resembles Darwinian evolution in cancers, which drives HCC behavioral and immunological heterogeneity. Research on lung cancer shows that non-heritable ITH, in contrast to heritable ITH, indicates phenotypic heterogeneity, and predicts ongoing process dynamics (53). Under the comparative topologies of dendrograms at the genomic and transcriptomic levels, functional ITH has been found to affect proliferative properties, epithelial or mesenchymal features, and clinical and histologic subtypes (53). Similarly, behavioral ITH affects the proliferation, invasiveness, and metabolic phenotypes of HCC. Immunological ITH, in contrast, refers to differences in immunogenicity, adjuvanticity, and immune escape, which determine whether or not tumor cells belong to heterobiotics, activate the immune response, and escape detection by the immune system. (54) Interestingly, the research applies multiregional genomic and immunological landscapes on a single HCC and displays a significant degree of immunological ITH in HCC, which is associated with tumoral transcriptomic ITH, especially malignancy and development. Mechanistically, immune stroma with augmented immunological ITH exhibits attenuated immune selective pressure, which confers on HCC cells the capacity to evade or suppress the immune system (55). Coincidentally, another publication has reported that multipoint biopsy sampling can completely restore ITH at both inheritable and non-heritable levels, helping us to understand the interactions between immunological ITH and tumor evolution (56). Histologic analysis of the different regions of HCC revealed immune infiltration in the abnormal tumor regions. In particular, HCC regions exhibiting transcriptomic ITH reveal the heterogeneity of tumor-infiltrating lymphocytes (TILs), mainly T-cell infiltrating *via* B- and T-cell receptor (B/TCR) RNA sequencing reads mapping to VDJ loci to assess the degree and characteristics of TIL burden (54). Moreover, the article concludes that passenger mutation, which offers no survival advantage, gathers more TILs than driver mutation, which confers a survival advantage (40, 54). Single-cell RNA sequencing (scRNA seq) and cytometry by time of flight (CyTOF) have been used to examine stromal TILs, especially some types of T cells, and their locations relative to cancer cells (57). T cells can be crudely classified as CD4+ or CD8+ T cells based on the type of glycoprotein found on the cell surface. Subsets of CD4+ cells comprise conventional T cells (Tconv cells), T follicular helper cells (TFH cells), regulatory T cells (Treg cells), dysfunctional T cells, naive T cells, effector T cells (TE cells), and memory T cells (TM cells) (**Table 1**). CD8+ T cells include naive T cells, TE cells, TM cells, and dysfunctional T cells, subdivided according to their level of differentiation (58, 59, 63–67). Dysfunctional T cells are described as dysfunctional or exhausted because they express a higher than usual number of inhibitory receptors such as the programmed cell death protein 1 (PD1) receptor, T-cell immunoglobulin mucin receptor 3 (TIM3), and receptors for lymphocyte activation gene 3 protein (LAG3), cytotoxic T lymphocyte-associated antigen 4 (CTLA4), CD200, and 2B4, and secrete lower than normal levels of cytokines such as tumor necrosis factor (TNF), interleukin 2 (IL-2), and interferon γ (IFNγ) (60–62). Even exhausted T cells demonstrate heterogeneity, but they are not found in all cancer types (68). However, studies of lung cancer and HCC cells have confirmed the presence of exhausted T cells with augmented expression of inhibitory receptors, including receptors for PD1 (encoded by the *PDCD1* gene), CTLA4, and LAG3 (65, 67).

**Table 1:** Types of immune cells in tumors and their functions.

Type of immune cell	Introduction	Reference
Tconv cell	Tconv cells promote tumor control through the stimulation of, among other cells, CD8+ T cells, natural killer (NK) cells, and a broad range of other innate immune cell types. Furthermore, they exert cytotoxic functions that result in the killing of human leukocyte antigen (HLA) class II-expressing tumor cells or they inhibit tumor growth through the secretion of IFNγ and TNF	(58)
TFH cell	The exact role of TFH cells in tumor immunity is unclear; these cells may contribute to the generation of tertiary lymphoid structures (TLSs) at the tumor site and thereby shape intratumoral CD8+ T-cell and B-cell responses	(59)
Treg cell	Treg cells can counteract tumor-specific immune responses by suppressing the infiltration and antitumor activity of, among other cells, CD8+ T cells and macrophages	(58)
Dysfunctional T cell	Dysfunctional T cells express increased inhibitory receptors such as programmed cell death protein 1 (PD1), T-cell immunoglobulin mucin receptor 3 (TIM3), lymphocyte activation gene 3 protein (LAG3), cytotoxic T lymphocyte-associated antigen 4 (CTLA4), CD200, and 2B4, together with reducing the secretion of cytokines such as TNF, IL-2, and IFNγ	(60–62)
Cytotoxic T lymphocyte	CTLs recognize and lyze target cells through the release of perforin and granzymes. CTLs are activated by DC antigen presentation *via* the major histocompatibility complex (MHC) class I antigen to the T-cell receptor. Apoptosis is induced in cells expressing a specific antigen	(63)
Naive T cell	Naive T cells are CD3+CD4+ and CD3+CD8+ cells that differentiate into effector T cells (CD4+ T helper cells or CD8+ cytotoxic T cells) in secondary lymphoid organs or TLSs after stimulation with three signals: antigen, co-stimulatory molecules, and cytokines	(63)
Memory T cell	Memory T cells are CD3+CD4+CD45RO+ and CD3+CD8+CD45RO+ cells that have encountered antigen and that respond to antigenic stimulation faster and with greater intensity than naive T cells	(63)

Intriguingly, scRNA seq has shown that, in contrast to CD8+ T cells, CD4+ T cells are not found in the stroma of all tumors. Unlike CD8+ T cells, which have a distinct role in cancers, which is to drive carcinomatosis, CD4+ T cells play varying roles in the process of tumor promotion (either oncogenesis or carcinomatosis) (69). It has been reported that CD4+ T-cell apoptosis, which can be induced by linoleic acid, which enhances the expression of carnitine palmitoyl transferase (CPT), accelerates the development of HCC (70). Other reports also confirm the inhibitory significance of CD4+ T cells in carcinogenesis and suggest that the presence of these cells is an independent prognostic marker in HCC (71, 72). These multiple features of T cells and their secretions significantly contribute to immunological ITH in HCC. Remarkably, immunological ITH is not a binary state; TILs in the tumoral stroma represent gradual, continuous, and highly plastic phenotypes for the varying TMEs. Traditionally, experts categorize TAMs found in stromal compartments as proinflammatory (M1-like) TAMs or anti-inflammatory (M2-like) TAMs, which is a relatively simple classification obscuring the complexity and plasticity of TAMs (18). The relative proportions of different TAM subpopulations and their cellular states, surface protein expression, and individual secretions account in large part for immunological ITH in HCC stroma (63, 73).

A well-known classification divides tumors into two categories based on their sensitivity to immunological therapy: “hot” tumors are generally sensitive to immunological therapy and contain abundant CD8+ cytotoxic T lymphocytes (CTLs), whereas “cold” tumors are significantly resistant to immunological treatment and contain limited numbers of CTLs (74). The progression and clinical stage of tumors are closely linked to the presence of CTLs, and the Immunoscore, a standardized method of quantification of CTLs in the TME, as a reflection of a tumor’s immunological state, has been validated as a measure of tumor aggressiveness and prognosis (74–77). Subsequently, a third immune profile, so-called “altered” states, was identified in colorectal cancer (CRC) in 2009 (78). The “altered” profile has since been split into two phenotypes: the “excluded” phenotype (reflecting the fact that CTLs are excluded at the edge of the invasive margin by the dense stroma) and the “immunosuppressed” phenotype (reflecting the fact that the tumor contains a small number of CTLs, which represent not a stromal barrier but an immunosuppressive TME) (79, 80).

The previous study on HCC, which integrated data from RNA-seq, TCR-seq, single-nucleotide polymorphism (SNP) array, and DNA-seq analyses across several regions in the same HCC specimen, illustrates a significant immunological ITH signature (54). The same patient (P02) exhibited diverse tumor states, including immune “hot” phenotypes with poor differentiation and immunogenicity (H2.a), immune “cold” phenotypes with good differentiation (H2.b, H2.c, and H2.d), and an intermediate phenotype (H2.e). The results agree with the previous concept that transcriptomic ITH is strongly associated with immunological ITH in HCC. Moreover, regional ITH in the same patient represents different clinical phenotypes and evolutional stages, reflecting spatiotemporal evolution. A better understanding of behavioral ITH and immunological ITH in HCC, and how ITH manipulates phenotypic arrangements under different TMEs, would contribute to the immunotherapy revolution and provide new directions in molecular treatment.

## Glycolysis in HCC

**Figure 3:**
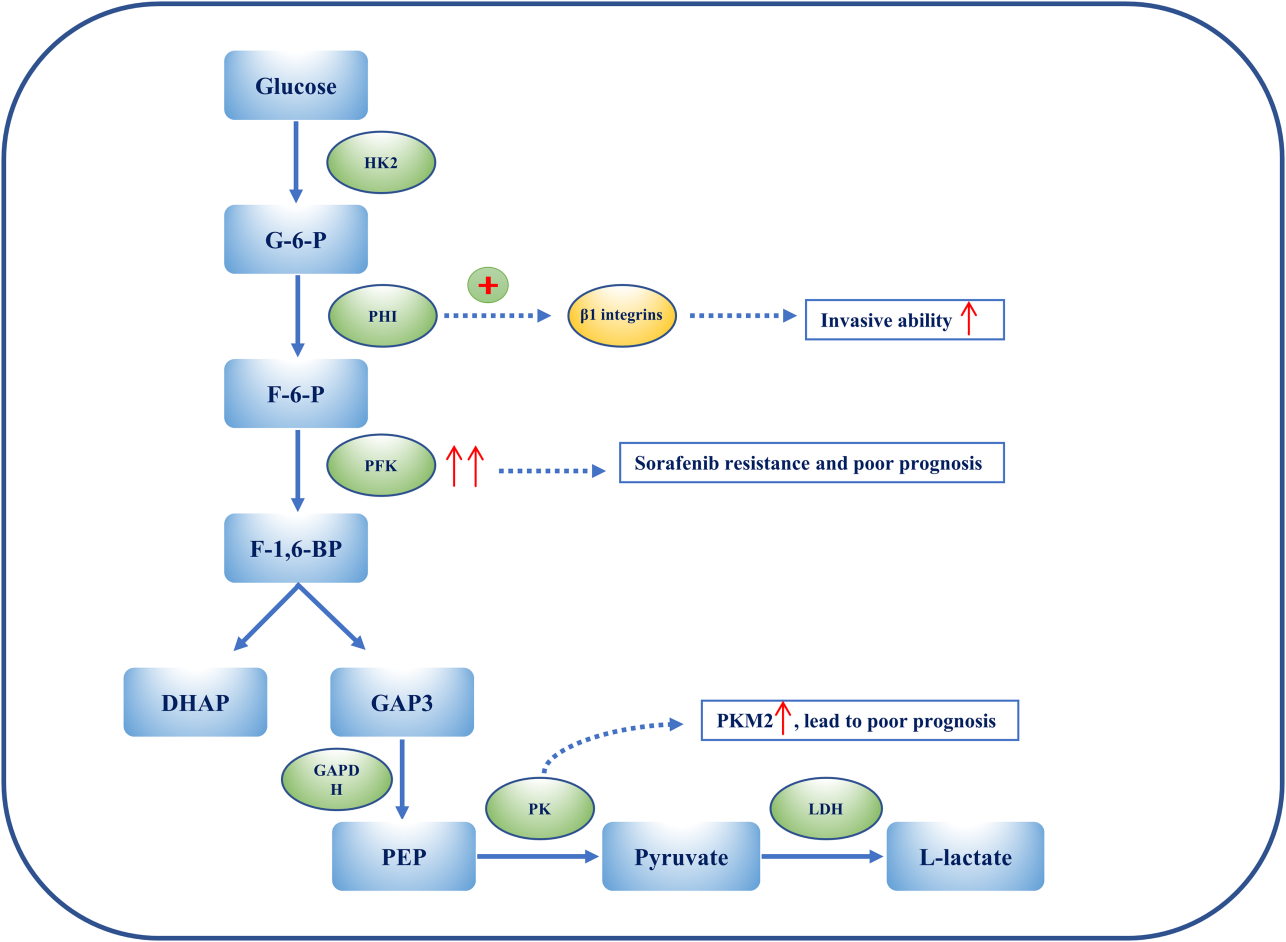
The heterogeneity of glycolysis in HCC. Metabolic ITH is exemplified by glycolysis in HCC. PHI can enhance the ability of HCC cell lines to penetrate Matrigel by activating β1 integrins lines. Overexpression of PFKFB3 results in sorafenib resistance, and elevated PFKFB3 is generally associated with poor outcomes and worse clinical manifestations in HCC. Among PKs, PKM2 is considerably up-regulated in HCC patients, and this is associated with a poor prognosis. Overexpression of PKM2 results in an increase in IFNγ-positive CD8+ T cells in the HCC mouse model by activating the immune checkpoint blockade.

Aerobic glycolysis was first proposed in rat liver cancer as the Warburg effect in the 1920s; researchers observed that cancer tissue consumes less oxygen (O_2_) than normal tissue. In other words, HCC converts glucose flux product (pyruvate) into lactate in the cytoplasm rather than transporting pyruvate into mitochondria for use in the Krebs cycle (**Figure 3**). This distinct feature of HCC is probably related to metabolic reprogramming and metabolic ITH. Of note, metabolic plasticity ensures that HCC has strong adaptability in nutrient-deprived TMEs through increasing scavenging pathways, including autophagy, apoptosis, and reverse Warburg effects associated with CAFs, generating intermediate products for metabolic utilization (81, 82). In general, aerobic glycolysis is essential during inception, growth, proliferation, invasion, and immune evasion. This paper promotes a better understanding of glycolysis and ITH in HCC, in particular its pathogenesis, detection, and molecular treatment.

Aggressive cells (such as HCC and pancreatic cancer cells) undergo extensive metabolic rewiring throughout their development. Metabolic reprogramming of glucose, lipid, and amino acids is found in both *in situ* and metastatic HCC; metabolites and Taylorism produced from reprogramming processes are necessary for energy production and anabolism, including cellular membranes, nucleotides, extracellular matrix (ECM), and cell cytoskeleton, which are fundamental for proliferation, invasion, and metastasis (83–86) (**Table 2**). Because glucose uptake is enhanced in HCC, the expression of glucose transporter 1 (GLUT-1), as a membrane channel for glucose, also increases. This unique feature, i.e., augmented glucose uptake, can be exploited to enable the early detection of HCC and its systemic metastases by positron emission tomography/computed tomography, by replacing glucose with fluorodeoxyglucose F 18 (^18^F-FDG), a glucose derivative used as a radiotracer. (96) The expression of GLUT-1 has been shown to be augmented in the stained membranes of HCC cells, and thus the level of expression of GLUT-1 could be used to evaluate liver lesions (87). In addition, miR-505 (micro-RNA-505) down-regulation of GLUT-1 expression attenuates glucose uptake and lactate generation in HCC cells. Together, miR-505 impairs HCC growth by inhibiting the expression of insulin-like growth factor 1 receptor (IGF-1R) and damaging glycolysis in HCC cells (88).

**Table 2:** Enzymes of glycolysis in HCC, their functions, and their impacts on therapy.

Enzymes	Introduction	Reference
GLUT-1	The expression of GLUT-1 is increased in HCC. It has been shown that GLUT-1 expression is augmented in in stained membranes from HCC, and GLUT-1 level could be used to evaluate liver lesions	(87)
miR-505	miR-505 down-regulation of GLUT-1 expression attenuates glucose uptake and lactate generation in HCC. miR-505 impairs HCC growth by inhibiting the expression of IGF-1R and damaging glycolysis in HCC cells	(88)
HK2	Silencing of HK2 leads to a reduction in glucose flux to pyruvate and lactate (glycolysis), but the tricarboxylic acid (TCA) cycle (OXPHOS) is unchanged. Depletion of HK2 synergistic with sorafenib sensitizes HCC cells to cell death and inhibits the mTORC1, together with metformin	(89)
PHI	PHI not only catalyzes reactions during glycolysis, but also acts as a cytokine, inducing invasion and metastasis. PHI binds to its receptor, Mr 78,000 glycoprotein (gp78), and promotes MMP-2 secretion, adhesion, and motility for enhanced invasive ability through Matrigel by activating β1 integrins in HCC cell lines	(90)
PFK	PFKFB3, having the strongest phosphofructo-2-kinase activity, is typically up-regulated in cancers including HCC. Overexpression of PFKFB3 and a high flux of glycolysis partly account for sorafenib resistance, which could be targeted by aspirin, as aspirin induces apoptosis together with sorafenib. Elevated levels of PFKFB3 is generally associated with poor outcomes and worse clinical manifestations; therefore, regulating PFKFB3 could not merely inhibit the activity of PFK to target glycolysis, but also arrest cell cycle and cell death in HCC	(91, 92)
GAPDH	Immunohistochemical staining of HCC tissue shows that GAPDH in the nucleus is positively associated with HIF-1α. Despite this, patients with overexpressed HIF-1α or low levels of GAPDH have lower OS and a poor prognosis	(93)
PK	Among PKs, PKM2 is notably up-regulated in HCC patients, and a high level of PKM2 is associated with a poor prognosis. Silencing of PKM2 inhibits HCC proliferation, migration, and invasion, whereas overexpression of PKM2 increases IFNγ-positive CD8+ T cells in the HCC mouse model by activating the immune checkpoint blockade	(94)
LDH	LDH could predict clinical outcomes, such as PFS and OS, in HCC patients treated with sorafenib	(95)

Hexokinase 2 (HK2) is an essential enzyme in aerobic glycolysis, catalyzing the conversion of glucose to glucose 6-phosphate (G-6-P). Silencing HK2 reduces glucose flux to pyruvate and lactate (glycolysis), but has no effect on the tricarboxylic acid (TCA) cycle (OXPHOS). Depletion of HK2, along with sorafenib, sensitizes HCC cells to cell death and inhibits the mammalian target of rapamycin (mTORC1), together with metformin (89). G-6-P is then converted to fructose 6-phosphate (F-6-P), a reaction that is catalyzed by phosphohexose isomerase (PHI), also known as autocrine motility factor (AMF), phosphoglucose isomerase (PGI), or glucose-6-phosphate isomerase (GPI). PHI (AMF) not only catalyzes reactions during glycolysis, but also acts as a cytokine, inducing invasion and metastasis. PHI binds to its receptor, Mr 78,000 glycoprotein (gp78), and promotes matrix metalloproteinase 2 (MMP-2) secretion, adhesion, and motility, thus enhancing the invasive ability of HCC cell lines, enabling them to penetrate Matrigel^®^ (Corning Life Sciences, Corning, NY, USA) by activating β1 integrins (90). Phosphofructokinase (PFK) converts F-6-P to fructose 1,6-biphosphate (F-1,6-BP), which is the second rate-limiting step in glycolysis. PFK is activated by fructose-2,6-biphosphate (F-2,6-BP), the most allosteric activator for PFK. Moreover, F-2,6-BP is catalyzed by phosphofructo-2-kinase/fructose-2,6-biphosphatase (PFK-2/PFKFB), which has two separate catalytic centers. Specifically, PFKFB3, having the strongest phosphofructo-2-kinase activity, is typically up-regulated in cancers, including HCC (91, 92). Overexpression of PFKFB3 and the high flux of glycolysis contribute to sorafenib resistance, and could be targeted by aspirin, as aspirin, in combination with sorafenib, induces apoptosis (91). Elevated PFKFB3 is generally associated with poor outcomes and worse clinical manifestations; therefore, regulating PFKFB3 could not merely inhibit the activity of PFK to target glycolysis, more than arrest cell cycle and cell death in HCC. F-1,6-BP subsequently decomposes into 3-phosphoglyceraldehyde (GA3P) and dihydroxyacetone phosphate (DHAP). Subsequently, a series of reactions catalyzed by enzymes, such as triose phosphate isomerase (TPI), glyceraldehyde 3-phosphate dehydrogenase (GAPDH), phosphoglycerate mutase (PGAM), and enolase, convert intermediates to produce phosphoenolpyruvate (PEP). Interestingly, GAPDH is generally used as a reference in quantitative reverse transcription–polymerase chain reaction (qRT-PCR) and Western blotting because of its stable expression during different states. However, immunohistochemical staining of HCC tissue shows that GAPDH in the nucleus is positively associated with the hypoxia-inducible factor (HIF-1α). Despite this, patients with overexpressed HIF-1α or low levels of GAPDH have a poor prognosis and low overall survival (OS) (93). Following this, PEP is catalyzed to pyruvate by pyruvate kinase (PK) and produces ATP, which is the last committed step in glycolysis. Among PKs, PKM2 is notably up-regulated in HCC patients and a high level of PKM2 is associated with a poor prognosis. Silencing PKM2 inhibits HCC proliferation, migration, and invasion whereas overexpression of PKM2 increases interferon gamma (IFNγ)-positive CD8+ T cells in the HCC mouse model by activating an immune checkpoint blockade (94). Intermediate F-1,6-BP is an allosteric activator of PKM2, whereas ATPacetyl-coenzyme A (CoA), and L-cysteine are allosteric inhibitors of PKM2 (97, 98). Shikonin is a specific inhibitor of PKM2, demonstrating carcinomatosis in HCC. In addition, shikonin In addition, shikonin promotes nuclear localization of PKM2 to recruit Nrf2, then activates BAG3 downstream of Nrf2 to provide a protective effect for cell survival (99). The final step in glycolysis is the conversion of pyruvate to L-lactate, catalyzed by lactate dehydrogenase (LDH). It has been suggested that the measurement of LDH could predict clinical outcomes such as progression-free survival (PFS) and OS in HCC patients treated with sorafenib (95).

A better understanding of glycolysis could not only shed light on the metabolic frame in HCC, but also uncover the relationship between metabolic ITH and immunological ITH, and its evolution in HCC. Indeed, metabolic ITH in aggressive cancers seems to regulate immunological ITH and promote phenotypic transformation. Notably, increased lactate dehydrogenase A (LDHA) is related to poor outcomes in HCC patients and is negatively associated with markers of CTLs. Moreover, lactate dehydrogenase A (LDHA)-related lactate accumulating in the TME creates an acidic stroma favoring the transport of substrates such as glucose and their uptake by HCC cells (100). It has consistently been found that lactate, together with an acidic microenvironment, suppresses some functions of immune cells and changes their morphology, thus reducing their survival. More precisely, lactic acid increases inhibitors’ immunosurveillance by attenuating the survival and function of T and NK cells, leading to the immune escape of tumor cells and a more malignant phenotype (101). Increasing evidence suggests that malignant cancers undergo metabolic adjustment in different TMEs to satisfy growth demands at every stage of the metastatic cascade. According to this view, metabolic flexibility (the use of the same metabolites at different stages of metastasis) and metabolic plasticity (the use of other metabolites that can satisfy identical demands during the metastatic cascade), which are notable properties of aggressive cancers, account for metabolic ITH in variable microenvironments and confer a growth advantage on aggressive cells when the TME is changeable (102). The metabolic profile reflects both the glycolytic phenotype (related to chemosensitivity and rapid proliferation) and the oxidative phenotype (associated with chemoresistance and late proliferation) coexist in glioblastoma, which definitively shows that metabolic ITH exists and is connected to the progression of aggressive cancers.

## Conclusion

To summarize, we have described the epidemiology of HCC and reported risk factors for HCC and worldwide morbidity. We then summarized ITH in HCC, including genetic ITH, epigenetic ITH, behavioral ITH, and immunological ITH. Of these, we mainly discussed metabolic ITH and immunological ITH and their role in the progression of ITH. Of note, metabolic ITH, particularly the glycolytic and oxidative phenotypes, is common in HCC. We hope that our findings will enable heterogeneity of the tumoral ecosystem and the resulting metabolic adaptation of tumor cells to be exploited in order to develop novel therapeutic approaches for HCC patients.

## Author contributions

F-QX and M-MD contributed equally to this work. F-QX and M-MD contributed to the conception and design of the study. F-QX and M-MD wrote the first draft of the manuscript. Z-FW and L-DC wrote sections of the manuscript and provided critical revisions. All authors contributed to the article and approved the submitted version.

## Glossary

**Table:**

^18^F-FDG	fluorodeoxyglucose F 18
AMF	autocrine motility factor
ATP	adenosine triphosphate
B/TCR	B- and T-cell receptor
CAF	cancer-associated fibroblast
CoA	coenzyme A
CPT	carnitine palmitoyltransferase
CRC	colorectal cancer
CTLA4	cytotoxic T lymphocyte-associated antigen 4
CyTOF	cytometry by time of flight
DC	dendritic cell
DHAP	dihydroxyacetone phosphate
ECM	extracellular matrix
F-1,6-BP	fructose 1,6-biphosphate
F-2,6-BP	fructose 2,6-biphosphate
F-6-P	fructose 6-phosphate
FDA	Food and Drug Administration
G-6-P	glucose 6-phosphate
GA3P	3-phosphoglyceraldehyde
GAPDH	glyceraldehyde-3-phosphate dehydrogenase
GLUT-1	glucose transporter 1
gp78	Mr 78,000 glycoprotein
GPI	glucose-6-phosphate isomerase
HBV	hepatitis B virus
HCC	hepatocellular carcinoma
HCV	hepatitis C virus
HIF-1α	hypoxia-inducible factor
HLA	human leukocyte antigen
HK2	hexokinase 2
HSC	hepatic stellate cell
ICC	intrahepatic cholangiocarcinoma
IFNγ	interferon γ
IGF-1R	insulin-like growth factor 1 receptor
IL-2	interleukin 2
ITH	intratumoral heterogeneity
LAG3	lymphocyte activation gene 3 protein
LDH	lactate dehydrogenase
MHC	major histocompatibility antigen
miR-505	micro-RNA-505
MMP-2	matrix metalloproteinase 2
mTORC1	mammalian target of rapamycin
NASH	non-alcoholic steatohepatitis
NGS	next-generation sequencing
OS	overall survival
OXPHOS	oxidative phosphorylation
PD1	programmed cell death protein 1
PEP	phosphoenolpyruvate
PFK	phosphofructokinase
PFK-2/PFKFB	phosphofructo-2-kinase/fructose-2,6-biphosphatase
PFS	progression-free survival
PGAM	phosphoglycerate mutase
PGI	phosphoglucose isomerase
PHI	phosphohexose isomerase
PK	pyruvate kinase
qRT-PCR	quantitative reverse transcription–polymerase chain reaction
scRNA	scRNA-seq: single-cell RNA sequencing
SNP	single-nucleotide polymorphism
TAM	tumor-associated macrophage
TCA	tricarboxylic acid
Tconv cell	conventional T cell
TE cell	effective T cell
TFH cell	T follicular helper cell
TIL	tumor-infiltrating lymphocyte
TIM3	T-cell immunoglobulin mucin receptor 3
TKI	tyrosine kinase inhibitor
TLS	tertiary lymphoid structure
TM cells	memory T cells
TME	tumor microenvironment
TNF	tumor necrosis factor
Treg cell	T regulatory cell
TPI	triose phosphate isomerase
UQCC3	ubiquinol–cytochrome c reductase complex assembly factor 3

## References

[1] Siegel RL Miller KD Fuchs HE Jemal A. Cancer statistics, 2021. CA Cancer J Clin (2021) 71(1):7–33. doi: 10.3322/caac.21654 33433946

[2] Bray F Ferlay J Soerjomataram I Siegel RL Torre LA Jemal A. Global cancer statistics 2018: GLOBOCAN estimates of incidence and mortality worldwide for 36 cancers in 185 countries. CA Cancer J Clin (2018) 68(6):394–424. doi: 10.3322/caac.21492 30207593

[3] Stavraka C Rush H Ross P. Combined hepatocellular cholangiocarcinoma (cHCC-CC): an update of genetics, molecular biology, and therapeutic interventions. J Hepatocell Carcinoma (2019) 6:11–21. doi: 10.2147/JHC.S159805 30643759 PMC6312394

[4] Yu MC Yuan JM. Environmental factors and risk for hepatocellular carcinoma. Gastroenterology (2004) 127(5 Suppl 1):S72–8. doi: 10.1016/j.gastro.2004.09.018 15508106

[5] Ascha MS Hanouneh IA Lopez R Tamimi TA Feldstein AF Zein NN. The incidence and risk factors of hepatocellular carcinoma in patients with nonalcoholic steatohepatitis. Hepatology (2010) 51(6):1972–8. doi: 10.1002/hep.23527 20209604

[6] Lee M Ko H Yun M. Cancer metabolism as a mechanism of treatment resistance and potential therapeutic target in hepatocellular carcinoma. Yonsei Med J (2018) 59(10):1143–9. doi: 10.3349/ymj.2018.59.10.1143 PMC6240564 30450847

[7] Sia D Villanueva A Friedman SL Llovet JM. Liver cancer cell of origin, molecular class, and effects on patient prognosis. Gastroenterology (2017) 152(4):745–61. doi: 10.1053/j.gastro.2016.11.048 PMC12160040 28043904

[8] Connell LC Harding JJ Abou-Alfa GK. Advanced hepatocellular cancer: the current state of future research. Curr Treat Opt Oncol (2016) 17(8):43. doi: 10.1007/s11864-016-0415-3 27344158

[9] Vitale I Sistigu A Manic G Rudqvist NP Trajanoski Z Galluzzi L. Mutational and antigenic landscape in tumor progression and cancer immunotherapy. Trends Cell Biol (2019) 29(5):396–416. doi: 10.1016/j.tcb.2019.01.003 30765144

[10] Wang J Cazzato E Ladewig E Frattini V Rosenbloom DI Zairis S. Clonal evolution of glioblastoma under therapy. Nat Genet (2016) 48(7):768–76. doi: 10.1038/ng.3590 PMC5627776 27270107

[11] Flavahan WA Gaskell E Bernstein BE. Epigenetic plasticity and the hallmarks of cancer. Science (2017) 357(6348). doi: 10.1126/science.aal2380 PMC5940341 28729483

[12] Vitale I Shema E Loi S Galluzzi L. Intratumoral heterogeneity in cancer progression and response to immunotherapy. Nat Med (2021) 27(2):212–24. doi: 10.1038/s41591-021-01233-9 33574607

[13] Costa A Kieffer Y Scholer-Dahirel A Pelon F Bourachot B Cardon M. Fibroblast heterogeneity and immunosuppressive environment in human breast cancer. Cancer Cell (2018) 33(3):463–79.e10. doi: 10.1016/j.ccell.2018.01.011 29455927

[14] Aoki T Chong LC Takata K Milne K Hav M Colombo A. Single-cell transcriptome analysis reveals disease-defining T-cell subsets in the tumor microenvironment of classic Hodgkin lymphoma. Cancer Discovery (2020) 10(3):406–21. doi: 10.1158/2159-8290.CD-19-0680 31857391

[15] Rosenthal R Cadieux EL Salgado R Bakir MA Moore DA Hiley CT. Neoantigen-directed immune escape in lung cancer evolution. Nature (2019) 567(7749):479–85. doi: 10.1038/s41586-019-1032-7 PMC6954100 30894752

[16] Zhang AW McPherson A Milne K Kroeger DR Hamilton PT Miranda A. Interfaces of malignant and immunologic clonal dynamics in ovarian cancer. Cell (2018) 173(7):1755–69.e22. doi: 10.1016/j.cell.2018.03.073 29754820

[17] Cao L Wu J Qu X Sheng J Cui M Liu S. Glycometabolic rearrangements–aerobic glycolysis in pancreatic cancer: causes, characteristics and clinical applications. J Exp Clin Cancer Res (2020) 39(1):267. doi: 10.1186/s13046-020-01765-x 33256814 PMC7708116

[18] Lambrechts D Wauters E Boeckx B Aibar S Nittner D Burton O. Phenotype molding of stromal cells in the lung tumor microenvironment. Nat Med (2018) 24(8):1277–89. doi: 10.1038/s41591-018-0096-5 29988129

[19] Neftel C Laffy J Filbin MG Hara T Shore ME Rahme GJ. An integrative model of cellular states, plasticity, and genetics for glioblastoma. Cell (2019) 178(4):835–49.e21. doi: 10.1016/j.cell.2019.06.024 31327527 PMC6703186

[20] Andor N Maley CC Ji HP. Genomic instability in cancer: Teetering on the limit of tolerance. Cancer Res (2017) 77(9):2179–85. doi: 10.1158/0008-5472.CAN-16-1553 PMC5413432 28432052

[21] Lin DC Mayakonda A Dinh HQ Huang P Lin L Liu X. Genomic and epigenomic heterogeneity of hepatocellular carcinoma. Cancer Res (2017) 77(9):2255–65. doi: 10.1158/0008-5472.CAN-16-2822 PMC5413372 28302680

[22] Warburg O Minami S. Versuche an Überlebendem carcinom-gewebe. Klinische Wochenschrift (1923) 2(17):776–7. doi: 10.1007/BF01712130

[23] Gatenby RA Gawlinski ET. The glycolytic phenotype in carcinogenesis and tumor invasion: Insights through mathematical models. Cancer Res (2003) 63(14):3847–54. 12873971

[24] Gatenby RA Gillies RJ. Why do cancers have high aerobic glycolysis? Nat Rev Cancer (2004) 4(11):891–9. doi: 10.1038/nrc1478 15516961

[25] Bose S Le A. Glucose metabolism in cancer. Adv Exp Med Biol (2018) 1063:3–12. doi: 10.1007/978-3-319-77736-8_1 29946772

[26] Pavlides S Tsirigos A Vera I Flomenberg N Frank PG Casimiro MC. Transcriptional evidence for the "Reverse warburg effect" in human breast cancer tumor stroma and metastasis: Similarities with oxidative stress, inflammation, alzheimer's disease, and "Neuron-glia metabolic coupling". Aging (Albany NY). (2010) 2(4):185–99. doi: 10.18632/aging.100134 PMC2881509 20442453

[27] Yang Y Zhang G Guo F Li Q Luo H Shu Y. Mitochondrial UQCC3 modulates hypoxia adaptation by orchestrating OXPHOS and glycolysis in hepatocellular carcinoma. Cell Rep (2020) 33(5):108340. doi: 10.1016/j.celrep.2020.108340 33147459

[28] Bencze G Bencze S Rivera KD Watson JD Hidvegi M Orfi L. Mito-oncology agent: fermented extract suppresses the warburg effect, restores oxidative mitochondrial activity, and inhibits *in vivo* tumor growth. Sci Rep (2020) 10(1):14174. doi: 10.1038/s41598-020-71118-3 32843660 PMC7447799

[29] Satriano L Lewinska M Rodrigues PM Banales JM Andersen JB. Metabolic rearrangements in primary liver cancers: Cause and consequences. Nat Rev Gastroenterol Hepatol (2019) 16(12):748–66. doi: 10.1038/s41575-019-0217-8 31666728

[30] Zellmer VR Zhang S. Evolving concepts of tumor heterogeneity. Cell Biosci (2014) 4:69. doi: 10.1186/2045-3701-4-69 25937891 PMC4417538

[31] Hajdu SI. A note from history: The first tumor pathologist. Ann Clin Lab Sci (2004) 34(3):355–6. 15487713

[32] Gaiti F Chaligne R Gu H Brand RM Kothen-Hill S Schulman RC. Epigenetic evolution and lineage histories of chronic lymphocytic leukaemia. Nature (2019) 569(7757):576–80. doi: 10.1038/s41586-019-1198-z PMC6533116 31092926

[33] McGranahan N Swanton C. Clonal heterogeneity and tumor evolution: Past, present, and the future. Cell (2017) 168(4):613–28. doi: 10.1016/j.cell.2017.01.018 28187284

[34] Hanahan D Weinberg RA. Hallmarks of cancer: the next generation. Cell (2011) 144(5):646–74. doi: 10.1016/j.cell.2011.02.013 21376230

[35] Flintoft L. Explaining aneuploidy patterns. Nat Rev Genet (2013) 14(12):825–. doi: 10.1038/nrg3634

[36] Jamal-Hanjani M Wilson GA McGranahan N Birkbak NJ Watkins TBK Veeriah S. Tracking the evolution of non-Small-Cell lung cancer. N Engl J Med (2017) 376(22):2109–21. doi: 10.1056/NEJMoa1616288 28445112

[37] Raynaud F Mina M Tavernari D Ciriello G. Pan-cancer inference of intra-tumor heterogeneity reveals associations with different forms of genomic instability. PloS Genet (2018) 14(9):e1007669. doi: 10.1371/journal.pgen.1007669 30212491 PMC6155543

[38] Salmon H Remark R Gnjatic S Merad M. Host tissue determinants of tumour immunity. Nat Rev Cancer (2019) 19(4):215–27. doi: 10.1038/s41568-019-0125-9 PMC7787168 30867580

[39] Teixeira VH Pipinikas CP Pennycuick A Lee-Six H Chandrasekharan D Beane J. Deciphering the genomic, epigenomic, and transcriptomic landscapes of pre-invasive lung cancer lesions. Nat Med (2019) 25(3):517–25. doi: 10.1038/s41591-018-0323-0 PMC7614970 30664780

[40] Hu Z Li Z Ma Z Curtis C. Multi-cancer analysis of clonality and the timing of systemic spread in paired primary tumors and metastases. Nat Genet (2020) 52(7):701–8. doi: 10.1038/s41588-020-0628-z PMC7343625 32424352

[41] Keenan TE Burke KP Van Allen EM. Genomic correlates of response to immune checkpoint blockade. Nat Med (2019) 25(3):389–402. doi: 10.1038/s41591-019-0382-x 30842677 PMC6599710

[42] Juric D Castel P Griffith M Griffith OL Won HH Ellis H. Convergent loss of PTEN leads to clinical resistance to a PI(3)Kα inhibitor. Nature (2015) 518(7538):240–4. doi: 10.1038/nature13948 PMC4326538 25409150

[43] Andor N Graham TA Jansen M Xia LC Aktipis CA Petritsch C. Pan-cancer analysis of the extent and consequences of intratumor heterogeneity. Nat Med (2016) 22(1):105–13. doi: 10.1038/nm.3984 PMC4830693 26618723

[44] Morris LG Riaz N Desrichard A Şenbabaoğlu Y Hakimi AA Makarov V. Pan-cancer analysis of intratumor heterogeneity as a prognostic determinant of survival. Oncotarget (2016) 7(9):10051–63. doi: 10.18632/oncotarget.7067 PMC4891103 26840267

[45] Patten DK Corleone G Győrffy B Perone Y Slaven N Barozzi I. Enhancer mapping uncovers phenotypic heterogeneity and evolution in patients with luminal breast cancer. Nat Med (2018) 24(9):1469–80. doi: 10.1038/s41591-018-0091-x PMC6130800 30038216

[46] Esteller M. Cancer epigenomics: DNA methylomes and histone-modification maps. Nat Rev Genet (2007) 8(4):286–98. doi: 10.1038/nrg2005 17339880

[47] Yu DH Waterland RA Zhang P Schady D Chen MH Guan Y. Targeted p16(Ink4a) epimutation causes tumorigenesis and reduces survival in mice. J Clin Invest (2014) 124(9):3708–12. doi: 10.1172/JCI76507 PMC4151219 25061879

[48] Prada D González R Sánchez L Castro C Fabián E Herrera LA. Satellite 2 demethylation induced by 5-azacytidine is associated with missegregation of chromosomes 1 and 16 in human somatic cells. Mutat Res (2012) 729(1-2):100–5. doi: 10.1016/j.mrfmmm.2011.10.007 22032830

[49] Bolouri H. Network dynamics in the tumor microenvironment. Semin Cancer Biol (2015) 30:52–9. doi: 10.1016/j.semcancer.2014.02.007 24582766

[50] Buczak K Ori A Kirkpatrick JM Holzer K Dauch D Roessler S. Spatial tissue proteomics quantifies inter- and intratumor heterogeneity in hepatocellular carcinoma (HCC). Mol Cell Proteomics (2018) 17(4):810–25. doi: 10.1074/mcp.RA117.000189 PMC5880102 29363612

[51] Furuta M Ueno M Fujimoto A Hayami S Yasukawa S Kojima F. Whole genome sequencing discriminates hepatocellular carcinoma with intrahepatic metastasis from multi-centric tumors. J Hepatol (2017) 66(2):363–73. doi: 10.1016/j.jhep.2016.09.021 27742377

[52] Zhang Q Lou Y Yang J Wang J Feng J Zhao Y. Integrated multiomic analysis reveals comprehensive tumour heterogeneity and novel immunophenotypic classification in hepatocellular carcinomas. Gut (2019) 68(11):2019–31. doi: 10.1136/gutjnl-2019-318912 PMC6839802 31227589

[53] Sharma A Merritt E Hu X Cruz A Jiang C Sarkodie H. Non-genetic intra-tumor heterogeneity is a major predictor of phenotypic heterogeneity and ongoing evolutionary dynamics in lung tumors. Cell Rep (2019) 29(8):2164–74.e5. doi: 10.1016/j.celrep.2019.10.045 31747591 PMC6952742

[54] Losic B Craig AJ Villacorta-Martin C Martins-Filho SN Akers N Chen X. Intratumoral heterogeneity and clonal evolution in liver cancer. Nat Commun (2020) 11(1):291. doi: 10.1038/s41467-019-14050-z 31941899 PMC6962317

[55] Nguyen PHD Ma S Phua CZJ Kaya NA Lai HLH Lim CJ. Intratumoural immune heterogeneity as a hallmark of tumour evolution and progression in hepatocellular carcinoma. Nat Commun (2021) 12(1):227. doi: 10.1038/s41467-020-20171-7 33431814 PMC7801667

[56] McGranahan N Swanton C. Cancer evolution constrained by the immune microenvironment. Cell (2017) 170(5):825–7. doi: 10.1016/j.cell.2017.08.012 28841415

[57] Zhang L Yu X Zheng L Zhang Y Li Y Fang Q. Lineage tracking reveals dynamic relationships of T cells in colorectal cancer. Nature (2018) 564(7735):268–72. doi: 10.1038/s41586-018-0694-x 30479382

[58] Ahrends T Borst J. The opposing roles of CD4(+) T cells in anti-tumour immunity. Immunology (2018) 154(4):582–92. doi: 10.1111/imm.12941 PMC6050207 29700809

[59] Qin L Waseem TC Sahoo A Bieerkehazhi S Zhou H Galkina EV. Insights into the molecular mechanisms of T follicular helper-mediated immunity and pathology. Front Immunol (2018) 9:1884. doi: 10.3389/fimmu.2018.01884 30158933 PMC6104131

[60] Wherry EJ Blattman JN Murali-Krishna K van der Most R Ahmed R. Viral persistence alters CD8 T-cell immunodominance and tissue distribution and results in distinct stages of functional impairment. J Virol (2003) 77(8):4911–27. doi: 10.1128/JVI.77.8.4911-4927.2003 PMC152117 12663797

[61] Baitsch L Baumgaertner P Devêvre E Raghav SK Legat A Barba L. Exhaustion of tumor-specific CD8^+^ T cells in metastases from melanoma patients. J Clin Invest (2011) 121(6):2350–60. doi: 10.1172/JCI46102 PMC3104769 21555851

[62] Thommen DS Koelzer VH Herzig P Roller A Trefny M Dimeloe S. A transcriptionally and functionally distinct PD-1(+) CD8(+) T cell pool with predictive potential in non-small-cell lung cancer treated with PD-1 blockade. Nat Med (2018) 24(7):994–1004. doi: 10.1038/s41591-018-0057-z 29892065 PMC6110381

[63] van der Leun AM Thommen DS Schumacher TN. CD8(+) T cell states in human cancer: insights from single-cell analysis. Nat Rev Cancer (2020) 20(4):218–32. doi: 10.1038/s41568-019-0235-4 PMC7115982 32024970

[64] Sade-Feldman M Yizhak K Bjorgaard SL Ray JP de Boer CG Jenkins RW. Defining T cell states associated with response to checkpoint immunotherapy in melanoma. Cell (2018) 175(4):998–1013.e20. doi: 10.1016/j.cell.2018.10.038 30388456 PMC6641984

[65] Guo X Zhang Y Zheng L Zheng C Song J Zhang Q. Global characterization of T cells in non-small-cell lung cancer by single-cell sequencing. Nat Med (2018) 24(7):978–85. doi: 10.1038/s41591-018-0045-3 29942094

[66] Tirosh I Izar B Prakadan SM Wadsworth MH 2nd Treacy D Trombetta JJ. Dissecting the multicellular ecosystem of metastatic melanoma by single-cell RNA-seq. Science (2016) 352(6282):189–96. doi: 10.1126/science.aad0501 PMC4944528 27124452

[67] Zheng C Zheng L Yoo JK Guo H Zhang Y Guo X. Landscape of Infiltrating T Cells in Liver Cancer Revealed by Single-Cell Sequencing. Cell. (2017) 169(7):1342–56.e16. 28622514 10.1016/j.cell.2017.05.035

[68] Li H van der Leun AM Yofe I Lubling Y Gelbard-Solodkin D van Akkooi ACJ. Dysfunctional CD8 T cells form a proliferative, dynamically regulated compartment within human melanoma. Cell (2019) 176(4):775–89.e18. doi: 10.1016/j.cell.2018.11.043 30595452 PMC7253294

[69] Kim HJ Cantor H. CD4 T-cell subsets and tumor immunity: The helpful and the not-so-helpful. Cancer Immunol Res (2014) 2(2):91–8. doi: 10.1158/2326-6066.CIR-13-0216 24778273

[70] Brown ZJ Fu Q Ma C Kruhlak M Zhang H Luo J. Carnitine palmitoyltransferase gene upregulation by linoleic acid induces CD4(+) T cell apoptosis promoting HCC development. Cell Death Dis (2018) 9(6):620. doi: 10.1038/s41419-018-0687-6 29795111 PMC5966464

[71] Li J Zhou J Kai S Wang C Wang D Jiang J. Functional and clinical characterization of tumor-infiltrating T cell subpopulations in hepatocellular carcinoma. Front Genet (2020) 11:586415. doi: 10.3389/fgene.2020.586415 33133170 PMC7561438

[72] Ma C Kesarwala AH Eggert T Medina-Echeverz J Kleiner DE Jin P. NAFLD causes selective CD4(+) T lymphocyte loss and promotes hepatocarcinogenesis. Nature (2016) 531(7593):253–7. doi: 10.1038/nature16969 PMC4786464 26934227

[73] Mantovani A Marchesi F Malesci A Laghi L Allavena P. Tumour-associated macrophages as treatment targets in oncology. Nat Rev Clin Oncol (2017) 14(7):399–416. doi: 10.1038/nrclinonc.2016.217 28117416 PMC5480600

[74] Galon J Costes A Sanchez-Cabo F Kirilovsky A Mlecnik B Lagorce-Pagès C. Type, density, and location of immune cells within human colorectal tumors predict clinical outcome. Science (2006) 313(5795):1960–4. doi: 10.1126/science.1129139 17008531

[75] Mlecnik B Tosolini M Kirilovsky A Berger A Bindea G Meatchi T. Histopathologic-based prognostic factors of colorectal cancers are associated with the state of the local immune reaction. J Clin Oncol (2011) 29(6):610–8. doi: 10.1200/JCO.2010.30.5425 21245428

[76] Pagès F Mlecnik B Marliot F Bindea G Ou FS Bifulco C. International validation of the consensus immunoscore for the classification of colon cancer: a prognostic and accuracy study. Lancet (2018) 391(10135):2128–39. doi: 10.1016/S0140-6736(18)30789-X 29754777

[77] Galon J Angell HK Bedognetti D Marincola FM. The continuum of cancer immunosurveillance: prognostic, predictive, and mechanistic signatures. Immunity (2013) 39(1):11–26. doi: 10.1016/j.immuni.2013.07.008 23890060

[78] Camus M Tosolini M Mlecnik B Pagès F Kirilovsky A Berger A. Coordination of intratumoral immune reaction and human colorectal cancer recurrence. Cancer Res (2009) 69(6):2685–93. doi: 10.1158/0008-5472.CAN-08-2654 19258510

[79] Mlecnik B Bindea G Angell HK Sasso MS Obenauf AC Fredriksen T. Functional network pipeline reveals genetic determinants associated with in situ lymphocyte proliferation and survival of cancer patients. Sci Transl Med (2014) 6(228):228ra37. doi: 10.1126/scitranslmed.3007240 24648340

[80] Galon J Bruni D. Approaches to treat immune hot, altered and cold tumours with combination immunotherapies. Nat Rev Drug Discovery (2019) 18(3):197–218. doi: 10.1038/s41573-018-0007-y 30610226

[81] Duda P Janczara J McCubrey JA Gizak A Rakus D. The reverse warburg effect is associated with Fbp2-dependent Hif1α regulation in cancer cells stimulated by fibroblasts. Cells (2020) 9(1). doi: 10.3390/cells9010205 PMC7016812 31947613

[82] Wei R Cao J Yao S. Matrine promotes liver cancer cell apoptosis by inhibiting mitophagy and PINK1/Parkin pathways. Cell Stress Chaperones (2018) 23(6):1295–309. doi: 10.1007/s12192-018-0937-7 PMC6237690 30209783

[83] Zheng YL Li L Jia YX Zhang BZ Li JC Zhu YH. LINC01554-mediated glucose metabolism reprogramming suppresses tumorigenicity in hepatocellular carcinoma *via* downregulating PKM2 expression and inhibiting Akt/mTOR signaling pathway. Theranostics (2019) 9(3):796–810. doi: 10.7150/thno.28992 30809309 PMC6376468

[84] Sengupta D Cassel T Teng KY Aljuhani M Chowdhary VK Hu P. Regulation of hepatic glutamine metabolism by miR-122. Mol Metab (2020) 34:174–86. doi: 10.1016/j.molmet.2020.01.003 PMC7044666 32180557

[85] Cai LY Chen SJ Xiao SH Sun QJ Ding CH Zheng BN. Targeting p300/CBP attenuates hepatocellular carcinoma progression through epigenetic regulation of metabolism. Cancer Res (2021) 81(4):860–72. doi: 10.1158/0008-5472.CAN-20-1323 33361394

[86] Berndt N Eckstein J Heucke N Gajowski R Stockmann M Meierhofer D. Characterization of lipid and lipid droplet metabolism in human HCC. Cells (2019) 8(5). doi: 10.3390/cells8050512 PMC6562484 31137921

[87] Zimmerman RL Fogt F Burke M Murakata LA. Assessment of glut-1 expression in cholangiocarcinoma, benign biliary lesions and hepatocellular carcinoma. Oncol Rep (2002) 9(4):689–92. doi: 10.3892/or.9.4.689 12066193

[88] Ren L Yao Y Wang Y Wang S. MiR-505 suppressed the growth of hepatocellular carcinoma cells via targeting IGF-1R. Biosci Rep (2019) 39(7). doi: 10.1042/BSR20182442 PMC6603277 31160483

[89] DeWaal D Nogueira V Terry AR Patra KC Jeon SM Guzman G. Hexokinase-2 depletion inhibits glycolysis and induces oxidative phosphorylation in hepatocellular carcinoma and sensitizes to metformin. Nat Commun (2018) 9(1):446. 29386513 10.1038/s41467-017-02733-4 PMC5792493

[90] Torimura T Ueno T Kin M Harada R Nakamura T Kawaguchi T. Autocrine motility factor enhances hepatoma cell invasion across the basement membrane through activation of beta1 integrins. Hepatology (2001) 34(1):62–71. doi: 10.1053/jhep.2001.25546 11431735

[91] Li S Dai W Mo W Li J Feng J Wu L. By inhibiting PFKFB3, aspirin overcomes sorafenib resistance in hepatocellular carcinoma. Int J Cancer (2017) 141(12):2571–84. doi: 10.1002/ijc.31022 28857200

[92] Wu Z Wu J Zhao Q Fu S Jin J. Emerging roles of aerobic glycolysis in breast cancer. Clin Transl Oncol (2020) 22(5):631–46. doi: 10.1007/s12094-019-02187-8 31359335

[93] Gong Y Zou B Peng S Li P Zhu G Chen J. Nuclear GAPDH is vital for hypoxia-induced hepatic stellate cell apoptosis and is indicative of aggressive hepatocellular carcinoma behavior. Cancer Manag Res (2019) 11:4947–56. doi: 10.2147/CMAR.S202268 PMC6553950 31239764

[94] Li TE Wang S Shen XT Zhang Z Chen M Wang H. PKM2 drives hepatocellular carcinoma progression by inducing immunosuppressive microenvironment. Front Immunol (2020) 11:589997. doi: 10.3389/fimmu.2020.589997 33193421 PMC7606949

[95] Faloppi L Scartozzi M Bianconi M Svegliati Baroni G Toniutto P Giampieri R. The role of LDH serum levels in predicting global outcome in HCC patients treated with sorafenib: implications for clinical management. BMC Cancer (2014) 14:110. doi: 10.1186/1471-2407-14-110 24552144 PMC3930857

[96] Shi X Xing H Yang X Li F Yao S Congwei J. Comparison of PET imaging of activated fibroblasts and (18)F-FDG for diagnosis of primary hepatic tumours: a prospective pilot study. Eur J Nucl Med Mol Imaging (2021) 48(5):1593–603. doi: 10.1007/s00259-020-05070-9 33097975

[97] Park SH Ozden O Liu G Song HY Zhu Y Yan Y. SIRT2-mediated deacetylation and tetramerization of pyruvate kinase directs glycolysis and tumor growth. Cancer Res (2016) 76(13):3802–12. doi: 10.1158/0008-5472.CAN-15-2498 PMC4930699 27197174

[98] Nakatsu D Horiuchi Y Kano F Noguchi Y Sugawara T Takamoto I. L-cysteine reversibly inhibits glucose-induced biphasic insulin secretion and ATP production by inactivating PKM2. Proc Natl Acad Sci U S A (2015) 112(10):E1067–76. doi: 10.1073/pnas.1417197112 PMC4364213 25713368

[99] Lu J Liu SY Zhang J Yang GM Gao GB Yu NN. Inhibition of BAG3 enhances the anticancer effect of shikonin in hepatocellular carcinoma. Am J Cancer Res (2021) 11(7):3575–93. PMC8332868 34354861

[100] Shiratori R Furuichi K Yamaguchi M Miyazaki N Aoki H Chibana H. Glycolytic suppression dramatically changes the intracellular metabolic profile of multiple cancer cell lines in a mitochondrial metabolism-dependent manner. Sci Rep (2019) 9(1):18699. doi: 10.1038/s41598-019-55296-3 31822748 PMC6904735

[101] Brand A Singer K Koehl GE Kolitzus M Schoenhammer G Thiel A. LDHA-associated lactic acid production blunts tumor immunosurveillance by T and NK cells. Cell Metab (2016) 24(5):657–71. doi: 10.1016/j.cmet.2016.08.011 27641098

[102] Bergers G Fendt SM. The metabolism of cancer cells during metastasis. Nat Rev Cancer (2021) 21(3):162–80. doi: 10.1038/s41568-020-00320-2 PMC8733955 33462499

